# Mouse single oocyte imaging by MALDI-TOF MS for lipidomics

**DOI:** 10.1007/s10616-020-00393-9

**Published:** 2020-04-09

**Authors:** Anna Bodzon-Kulakowska, Roberta Arena, Przemyslaw Mielczarek, Kinga Hartman, Paulina Kozoł, Ewa Gibuła-Tarlowska, Tomasz P. Wrobel, Łukasz Gąsior, Zbigniew Polański, Grazyna E. Ptak, Piotr Suder

**Affiliations:** 1 Department of Biochemistry and Neurobiology, Faculty of Materials Science and Ceramics, AGH University of Science and Technology, Cracow, Poland; 2 Institute of Genetics and Animal Breeding, Polish Academy of Sciences, Jastrzebiec, Poland; 3 Institute of Psychology, Jagiellonian University, Krakow, Poland; 4 Laboratory of Proteomics and Mass Spectrometry, Institute of Pharmacology, Polish Academy of Sciences, Krakow, Poland; 5 Institute of Nuclear Physics Polish Academy of Sciences, 31342 Krakow, Poland; 6 Malopolska Centre of Biotechnology, Jagiellonian University, Krakow, Poland; 7 Department of Genetics and Evolution, Institute of Zoology and Biomedical Research, Jagiellonian University, Krakow, Poland; 8 Department of Biomedical Sciences, University of Teramo, Teramo, Italy; 9 Department of Pharmacology and Pharmacodynamics, Medical University, Lublin, Poland; 10 Solaris National Synchrotron Radiation Centre, Jagiellonian University, Krakow, Poland

**Keywords:** MALDI-imaging, Single cell analysis, Mouse oocyte, Mink oocyte, Sample preparation methods

## Abstract

Reproductive cells are a very special kind of material for the analysis. Depending on the species, their dimensions allow for the application of mass spectrometry imaging-based techniques to receive a reasonable data for interpretation of their condition without any additional sample preparation steps, except for typical sample preparation characteristic for IMS protocols. A comparison between lipid profiles of oocytes could answer the question of the overall quality of the cells in the function of time or conditions of storage. Even tiny differences in the lipid profiles, but still detectable by bioinformatic analysis, could be crucial for the estimation of the conditions of the cells in various stages of development or aging. In our study, MALDI-TOF/TOF MSI was used to analyze and visualize the single oocytes. We deposited the cells on the transparent indium-tin-oxide (ITO) glass and marked their positions, which allowed for the fast localization of the cells and precise laser targeting in the ion source. We also optimized the usage of different MALDI matrices and different approaches. The proposed way of measurement allows analyzing quite a significant quantity of oocytes in a reasonably short time. During the analysis, the lipid composition of the single cell was successfully estimated in a conventional usage of the MALDI ion source, and the localization of lipids was confirmed by imaging mass spectrometry (IMS) analysis. The observed quantity of the lipids allowed for the application of the LIFT™ technique to obtain MS/MS spectra sufficient for lipids’ unambiguous identification. We hope that our idea of the oocyte analysis will help to elucidate chemical changes that accompany different processes in which oocytes are involved. There could be such fascinating phenomena as the oocyte maturation, changes in the lipid components during their storage, and much more.

## Introduction

Although analytical techniques are improved continuously, the single cell analysis still stays an analytical challenge. However, in some studies, this type of analysis seems to be the only rational approach. The case of the germinal cell: the oocyte, is a good example. Here, we have to deal with the extremely tiny amount of unique material. Pooling a set of oocytes to increase the sensitivity of the measurements might not allow for the analysis of individual variability. Thus, the possibility of single cell analysis in the search of markers of maturation, cell damages, etc. is very desirable in this kind of research.

Mass spectrometry imaging (MSI) is a technique of choice, where the spatial distribution of various substances should be indicated. Usually, this kind of analysis is done on the tissue cross-section, but different surfaces and various types of imprints (Bodzon-Kulakowska et al. 2015) might be used as well. Two major advantages of the IMS techniques are:

1. Simultaneous detection of as many substances from the surface, as can be ionized in the chosen experimental conditions,
2. The possibility of discovering new, unexpected substances, playing a role in the analyzed phenomena, without the prior knowledge of their involvement in the observed process.

In this study, we decided to use the MALDI ion source for the analysis of mouse and mink oocytes lipid composition.

Lipids stored in the mammal’s oocytes are the important energy source for the egg, used during preimplantation development. Cytoplasmic lipid droplets composition and localization could vary, depending on the oocyte, and later the embryo development stage. Even the oocytes at the same maturation stage may differ significantly from each other.

The evaluation of oocyte lipid content has been used to complete the information about the oocyte quality that seems to be closely related to its lipid composition. Analysis of Leroy et al. based on the optical density of the cytoplasm of the living oocytes isolated freshly from the ovaries, revealed consistent differences in lipids content between individual cells (Leroy et al. 2005a). A comparison of such oocytes showed that the darker oocytes that had higher capabilities to undergo preimplantation development contain more lipids, which are more abundant in saturated stearic acid. Whereas, pale oocytes displayed lower developmental potential and had higher levels of oleic and linoleic acids (Leroy et al. 2005a; Kim et al. 2001). Matorras et al. (1998) reported that competent human oocytes had significantly higher concentrations of unsaturated fatty acids, particularly linoleic acid (C18:2) and oleic acid (C18:1), and lower concentrations of total saturated fatty acids than non-viable oocytes. Additionally, it was shown that the lipid composition of an unfertilized oocyte, two- and four-cell embryos, and blastocysts is different and is characteristic for the particular stage of development (Ferreira et al. 2012).

Judging from the many studies and experiments, there are a lot of extracellular factors that influence lipid metabolism in oocytes and, in consequence, may influence the quality of the embryos. First of all, the difference in lipid composition between the in vitro and in vivo cultured blastocyst was indicated (Ferreira et al. 2012). In vitro cultured blastocyst possesses a more reproducible lipid profile, probably due to a more controlled environment during the development. Additionally, it was shown that different parameters of the cell culture might evoke changes in lipid composition, which may be very important for the embryo developmental success, as well as for its cryosensitivity. The group of Leroy et al. (Leroy et al. 2005b) showed in another study that addition of palmitic acid and stearic acid to the cell culture medium results in the negative effect on the progression of meiosis, and thus exert the effect on maturation, fertilization, cleavage rate, and blastocyst yield. Lapa et al. (2011) showed that in vitro maturation medium supplemented with trans-10 cis-12 conjugated linoleic acid may improve bovine oocyte competence.

Several studies indicated the important role of cumulus–oocyte complexes (COCs) for oocyte development in the context of lipids metabolism and transformations. In their works, Auclair et al. (2013), Dunning et al. (2014), and Sanchez-Lazo et al. (2014) indicated the role of cumulus cells in proper oocyte energy metabolism, especially in mitochondrial fatty acid β-oxidation. What is more, Lolicato et al. (2015) and Aardema et al. (2013), proved the protective role of the cumulus cell layer against the lipotoxic effect of free fatty acids whose increased level seems to be associated with female infertility (Jungheim et al. 2011).

This indicates the essential role of lipid content and composition in different aspects of oocyte biology (Prates et al. 2014).

The outbreak of rapid oocyte analysis took place in 2010 when for the first time, mass spectrometry was used for the analysis of single, intact oocytes from different species (bovine, sheep, fish, and fire ant) (Ferreira et al. 2010). This first study proved that it is possible to obtain reproducible and characteristic MALDI-MS fingerprints and measure the lipid composition of single oocytes from different species with no solvent extraction and additional, chemical manipulation. In the next study, DESI—another IMS ion source was shown to be able to measure the lipid components of oocytes as well as two- and four-cell embryos and blastocysts (Ferreira et al. 2012).

Afterward, analyses of oocytes were done up to date on targets as porcine oocytes (Pirro et al. 2014), bovine oocytes (ca. 175 um) (González-Serrano et al. 2013; Tata et al. 2013) bovine blastocysts (Leão et al. 2014; Gonçalves et al. 2016), *Bos Taurus Indicus* and *Bos Taurus Taurus* embryos (ca. 250 μm) (Sudano et al. 2012), or Zebrafish embryo (ca. 600 μm) (Dueñas et al. 2017). DESI-MS analysis was mainly used in this context. MALDI was used three times: twice in the standard setup, with the cells placed on the ground steel MALDI target plate, and once with the newly built, non-commercial arrangement (Dueñas et al. 2017). Lipid analysis by MALDI-MS provided valuable information about oocyte development and the influence of different circumstances on this process (Ferreira et al. 2010; Aardema et al. 2013; Ferreira et al. 2015; Silva-Santos et al. 2014). ToF–SIMS MS was also used for imaging of mouse oocytes, and analyses were done on freeze-dried cross sections of cells (Gulin et al. 2016). Obtained results were compared to AFM, SEM, and optical microscopic techniques. The review for ambient techniques could be found here (Ferreira et al. 2015).

Additionally, different, non-invasive techniques such as coherent anti-Stokes Raman scattering (CARS) microscopy could be used for the lipid quantification (Jasensky et al. 2016). Such techniques could be complementary to IMS since they allow for live-cell imaging, with higher resolution, but they lack lipid identification.

Determining the lipid composition of mouse oocytes may be particularly challenging due to the low lipid content, compared to other commonly used model mammalian species. An attempt based on a pooling of thousands of oocytes indicated that the mouse oocytes contain significantly smaller lipid content than oocytes of cattle, pigs, and sheep (Sturmey et al. 2009). Therefore, the estimation of the lipid composition of individual mouse oocytes requires possibly highly sensitive analytical equipment. MALDI-TOF MS seems to be the instrument of choice in such types of analyses.

Here we propose a fast method of oocytes screening analysis using the MALDI imaging approach. Placing the sample on the transparent ITO glass and marking its position by the marker (in our case nail polish, functionally similar to Tipp-EX used in other published experiments), allows for the fast localization of the cell in the ion source and precise laser targeting. This arrangement allows for a very fast analysis of a single cell. As a result, a significant number of cells could be measured in a reasonable time. It seems to be also a cheaper alternative to commercially available target plates dedicated to cell analyses.

Our methodology allows us to examine the oocytes from mice and minks, showing the ability to discriminate them, according to the differences in obtained MS spectra. This approach could also lead to more subtle analyses revealing a current condition or fertilization potential of oocytes populations, depending on time from isolation, conditions of storage, freezing media composition, or other factors. Additionally, we optimized MALDI imaging for the attempt to visualize a single oocyte. In our experiments, we tested:

1. The influence of washing the cells to remove the excess of the salt from the sample surface.
2. Matrix optimization for positive and negative ion modes.
3. Two ways of matrix deposition—using pipette and ImagePrep device (Bruker-Daltonics, Bremen, Germany).
4. LIFT fragmentation to identify lipids found in the samples.
5. Imaging experiment design to receive a reasonable result in the from the ion maps.

## Materials and methods

### Reagents

M2 culture medium, paraformaldehyde, PBS, polyvinylpyrrolidone, hexane, 2,5-dihydroxybenzoic acid (2,5-DHB), 9-aminoacridine, trifluoroacetic acid were obtained from Sigma-Aldrich, ultrapure methanol and water were obtained from Fluka, ethanol (96%) received from the local supplier.

### Experimental design

The aim of the study was to find the optimal way for the analysis of the lipid composition of quite a big number of oocytes (up to 25 items) during the single measurement. During the optimization process, the way of marking oocyte position was developed, several matrixes have been tested, and the best way of matrix deposition was elucidated. In the end, the image of the single cell was obtained.

### Collection of mouse and mink oocytes

All experiments on animals were done according to polish and EU law *(Polish Act on the Protection of Animals Used for Scientific or Educational Purposes. Pos. 266, 26.02.2015)* under the supervision and agreement of the Local Bioethics Committee.

Mice from C57BL/6 inbred strains and american minks (WT) were used throughout the experiments. Animal procedures were conducted at the Małopolska Center of Biotechnology; mice were obtained by the Institute of Zoology and Biomedical Research of Jagiellonian University (Krakow) while ovaries of american minks were provided by farms. Mice were maintained in a temperature- and light-controlled room (22 °C and 12 h light–dark cycle) and were provided with food and water ad libitum. Mice were ethically euthanized in the days just before the experiments. Oocytes were isolated with a standard procedure (e.g. Polanski et al. 2005). Briefly, the ovaries were dissected, placed in the drops of M2, common media for oocytes handling at RT, and punctured with the forceps tips to release the oocytes from the antral follicles. After mechanical removal of surrounding cumulus cells by gentle pipetting, the oocytes were collected for further processing. All oocytes handling procedures occurred under a stereomicroscope, Nikon SMZ 745 (Nikon, Tokyo, Japan). Oocytes were fixed in 4% paraformaldehyde for 20 min and washed, initially in 0.4% in PBS + 0,4% polyvinylpyrrolidone (PBS-PVP), then in Ultrapure water (Milli-Q, Millipore, Bedford, USA) in order to remove salts from the previous solution, and transferred on ITO glasses (Indium-Tin-Oxide glass, Bruker-Daltonics, Bremen, Germany). The ITO glass slides had been washed with hexane and ethanol in the ultrasonic bath, for 5 min each, just before use. The oocytes were placed through the use of a pneumatically-controlled pipette, ending with a pulled Pasteur pipette, which allows allocating the oocytes on the slide in a small volume of Ultrapure water (Milli-Q, Millipore, Bedford, USA), approximately 1 μl, which evaporates soon after sample allocation. The location of the dried oocytes on the slide was marked each time with a small dots done with nail polish. Eventual water excess was removed by placing the slides in the vacuum desiccator, and then the freshly prepared slides were stored at − 86 °C until assay.

### Oocyte localization on the ITO glass

To localize the oocytes in the UltrafleXtreme mass spectrometer’s camera, the position of every cell was marked by the three white dots painted directly on the glass under the magnification before matrix deposition. Dots were spotted 50–200 μm away from each cell, using white nail polish with the aid of fine brush consisted of three-four brush hairs. A picture of each oocyte with the markers has been taken before the matrix coating for easier cell localization during the measurements.

**Fig. 1:**
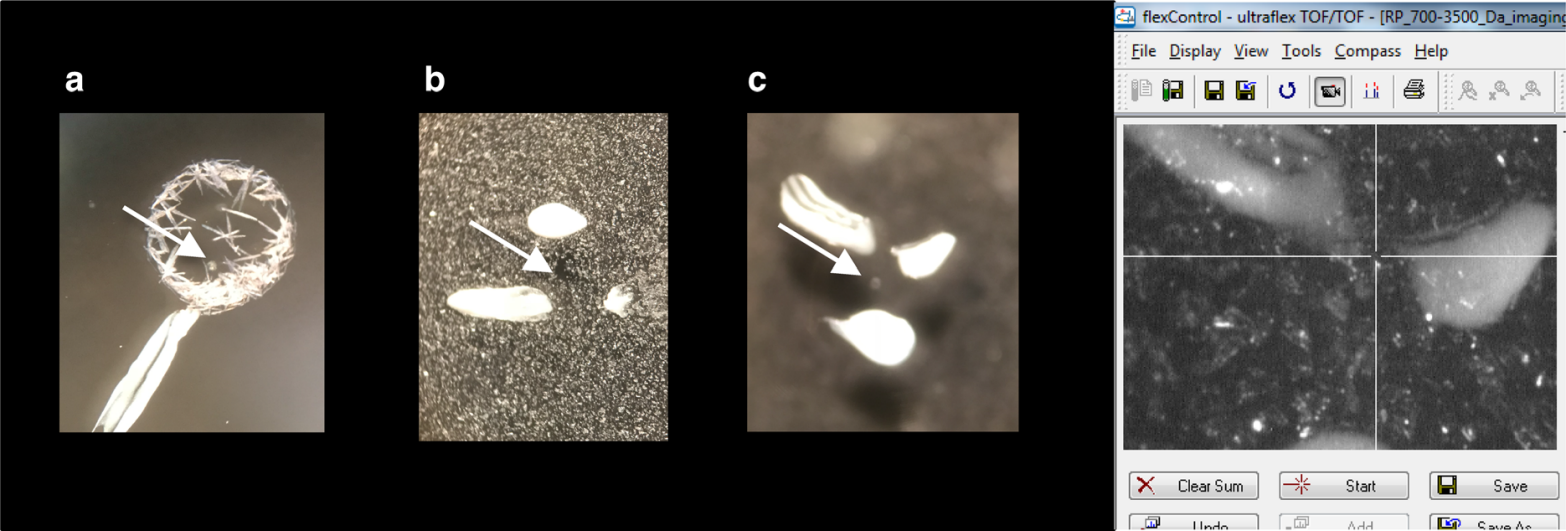
The view of the cell under the stereomicroscope and in the camera of the MALDI MS system. **a** The cell covered with the droplet of the DHB matrix (large crystals are visible). **b** The cell covered by the matrix with the aid of ImagePrep. **c** The picture of the cell prepared to be covered by the ImagePrep and its view in the MALDI system camera. The position of the cell is marked by the white dots of nail polish, which allowed for a quick finding of the cell

The exemplary cell is presented in Fig. 1. Such marking makes finding the cell on the ITO glass in the camera preview possible and relatively fast.

### Sample preparation for MALDI analysis (salt removal, matrix optimization and deposition)

The cells, stored at − 86 °C, were transferred just before the analysis to the desiccator and kept under the vacuum for about 30 min to exclude the water condensation during warming up to the room temperature.

Two ways of matrix deposition were tested during experiments: the classical “dried droplet approach,” where the drop (0.2 μl) of the matrix was placed over the analyzed cell, and the second approach, where the matrix is sprayed on the surface using ImagePrep (Bruker-Daltonics, Bremen, Germany). For the “dried droplet approach,” 150 mg/ml of 2,5-dihydroxybenzoic acid (2,5-DHB) matrix in methanol was prepared as recommended by Tata et al. (2013). Additionally, saturated α-Cyano-4-hydroxycinnamic acid (1:1, acetonitrile: H_2_O + 0.1% TFA, v/v/v) and the mixture of both (70 mg/ml DHB and CHCA 7:3, MetOH: H_2_O + 0.1% TFA, v/v/v) were tested. For the negative ion mode, 15 mg/ml of 9-aminoacridine dissolved in the ethanol–water solution (7:3, v/v), was used.

For ImagePrep, matrices were prepared according to the protocols recommended by the manufacturer. For positive ionization mode, DHB was used in a concentration of 30 mg/ml in methanol–water solution (1:1, v/v) acidified by trifluoroacetic acid (+ 0.2% v/v). For negative ion mode, 10 mg/ml of 9-aminoacridine dissolved in the ethanol–water solution (7:3, v/v) was used, without any additives. Samples were covered by the matrices using a standard protocol available for DHB.

### MALDI lipid analysis and identification

For analysis and imaging, the UltrafleXtreme MALDI-TOF/TOF instrument (Bruker-Daltonics, Bremen, Germany) was used. Before the analysis, ITO glasses with oocytes covered by the matrix were mounted on the standard glass slide holder. Conditions of the MS spectra acquisition were as follows: the scan range was set to m/z 300–3000. Laser power was usually set in a range of 18–25% of the maximum emission power. Matrix suppression was turned off. Holder’s random walk was limited to 200 μm diameter in a “partial sample” mode, with 500 shots at a raster spot. In a single cycle, 5000 laser shots were used for spectrum acquisition. To acquire the final spectrum from a single cell, which was localized with the aid of white dots from the nail polish, two cycles (equal to total 10,000 laser shots) were used. To receive a satisfactory resolution, the TOF analyzer was always set in the reflectron analysis mode. Calibration of the spectrometer was always performed directly before analysis using the Peptide Calibration Mixture provided by the spectrometer manufacturer. For lipid identification, fragmentation based on the LIFT technology was used. In every case, the manual selection of the parent ion was made.

### MALDI imaging of the single oocyte

Imaging was performed on the cell placed on the ITO glass. As nail polish (white dots positioning the cell localization) is able to generate a lot of peaks after laser irradiation without extensive contamination of the ion source, we usually set the imaging area covering a fragment of at least a single dot, closest to the cell. Such a region can serve as a positive control of the imaging process. The raster of the imaging area was set to 20 μm, which gave us a final image resolution of 1270 dpi. To avoid complete burn out of the imaged point on the cell before satisfactory spectrum acquisition, we used a “partial sample” random walk function. On every point of the raster nodes, the imaged area was also set to 20 μm. A random walk was set to move the laser in the new position inside the defined area after 500 laser shots and accumulate a spectrum from the total 5000 laser shots (10 points of spectrum acquisition for single raster node). A final resolution of the MS image was a compromise between spatial resolution and the capabilities of the instrument to receive reasonable spectra from the tiny area of the imaged biological material.

MS images were reconstructed in the FlexImaging software (Bruker-Daltonics, Bremen, Germany) dedicated for mass spectrometry imaging. Data from mass spectra and fragmentation spectra were interpreted with the aid of DataAnalysis and FlexAnalysis software of the same manufacturer.

### Statistical analysis

The spectra from the measurements of 12 oocytes from C57 mice and 7 minks were converted to an ASCII file. Preprocessing and statistical analysis of the MALDI spectra was performed in Matlab environment. Preprocessing included a baseline correction using a rubber band correction to user-defined points and normalization to unity for each analyzed region. Two groups of oocytes were assigned arbitrary class memberships (values of 2 and − 2), and Partial Least Squares Regression (PLS) was performed on selected regions, with a Leave-One-Out Cross-Validation (LOO-CV) scheme to determine the optimal number of latent variables (Lv) of the PLS model. This was done by finding the optimal CV Error, which in this case, was equal to 3 and corresponded to 97% of Y Variance.

## Results

At the beginning of the study, the DHB matrix was chosen for the measurement in the positive ion mode since it has the best s/n ratio and possesses the smallest number of additional signals from the matrix. Then, different ways of sample preparation were tested. First, as it was done in the study of Leão et al. (2014) and Sudano et al. (2012), the single cell was covered with the drop of the matrix (0.2 μl). Then matrix deposition with the ImagePrep device was tested. The second method was obviously better since small and evenly distributed crystals were formed. It is also a much more convenient method when we have to measure several cells on the same glass due to avoid variability during data acquisition and bias.

**Fig. 2:**
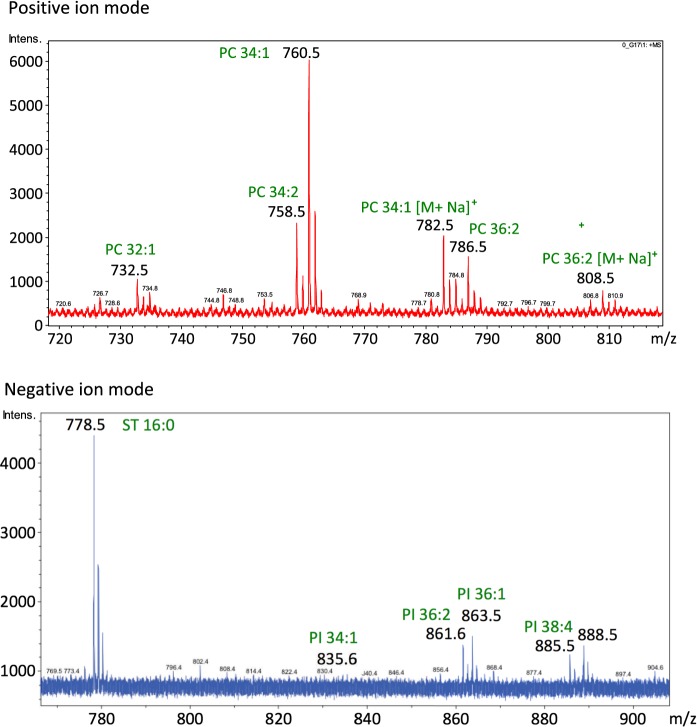
MALDI mass spectra from a single oocyte, measured in the positive and negative ion mode, in a range characteristic for lipid analysis (*PC* phosphocholines, *PI* phosphoinositols, *ST* sulfatides)

Below, mass spectra in the mass range specific for lipid analysis in the positive and negative ion mode, are presented in Fig. 2. Obtained peaks are in the form of protonated molecular ions and sodium adducts, characteristic for MALDI analysis.

MALDI-TOF analysis of the mouse oocytes showed the composition of the most abundant lipids present in the investigated cells. It was found out that the lipid quantity in the single oocyte is enough for the high-quality mass spectra and successful fragmentation of at least a few peaks. LIFT based fragmentation allowed for the unambiguous identification of fragmented molecules. In Table 1, the information about m/z characteristic for a single protonated, sodiated, or deprotonated molecule, with its identification, are included. In the analyzed cells mainly phosphocholines (PC), phospoinositols (PI), and sulfatides were observed. The number after abbreviation stands for the number of carbon atoms and double bonds in the fatty acyl chains at sn2 and sn3 positions summarily.

**Table 1:** Identification of characteristic peaks in positive and negative ion mode

Positive ion mode		Negative ion mode	
[m/z]	Identification	[m/z]	Identification
732.5	PC 32:1 [M+H]^+^	888.5	PI 38:4 [M−H]^−^
758.5	PC 34:2 [M+H]^+^	863.6	PI 36:1 [M−H]^−^
760.5	PC 34:1 [M+H]^+^	861.6	PI 36:2 [M−H]^−^
782.5	PC 34:1 [M+Na]^+^	835.6	PI 34:1 [M−H]^−^
786.5	PC 36:2 [M+H]^+^	778.5	ST 16:0 [M−H]^−^
808.5	PC 36:2 [M+Na]^+^		

The data about m/z and the form of ions (protonated molecular ion, or sodium adducts) are included (*PC* phosphocholines, *PI* phospoinositols, *ST* sulfatides)

**Fig. 3:**
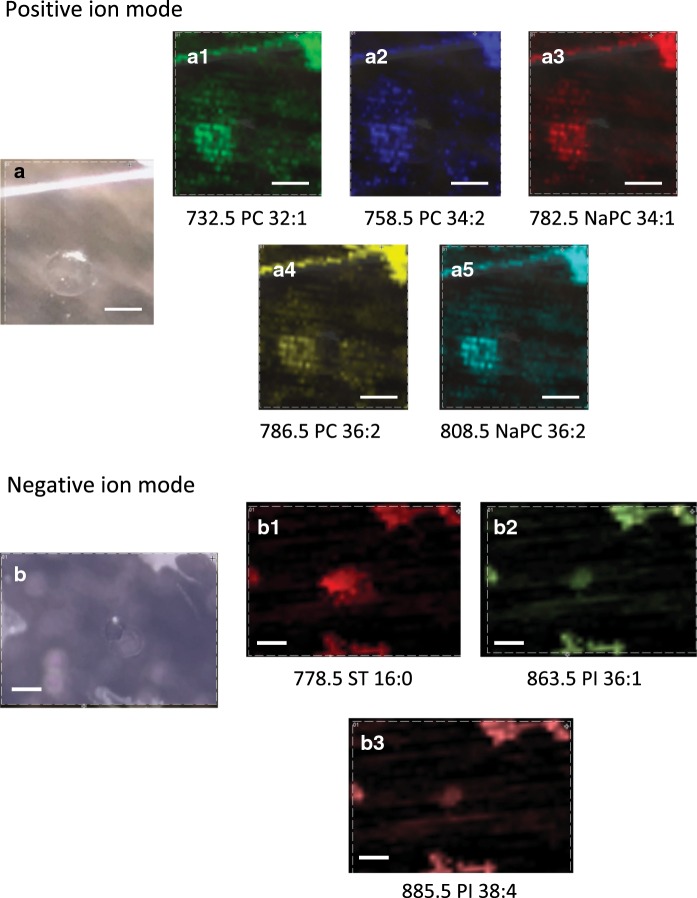
The examples of the results from MALDI imaging analysis of the single cell in the positive and negative ion modes. On the left, stereomicroscopic pictures of a single cell are presented (**a**, **b**). On the right, the ion maps for particular protonated/deprotonated ion and sodium adducts are presented (a1–5 positive ion mode, b1–b3 negative ion mode). Scale bar: 100 μm

The single oocyte with its surrounding was analyzed using MALDI imaging approach either in positive and negative ion mode. Using optimized analytical conditions, it was possible to visualize lipid distribution in a single oocyte (Fig. 3).

## Discussion

In this study, we presented quite a new approach for the analysis of single oocytes, which allows for both classical analysis of the lipid content of the cell, and for mass spectrometry imaging of the single cell, which adds a new interesting perspective for this kind of measurements. Below we discussed some tips and tricks based on obtained results, which could be helpful for anyone that would like to measure the lipid content of the single oocytes.

### Nail enamel as a localization marker

As it was previously mentioned, white nail polish was used to localize cells on the ITO glass surface in the camera of the mass spectrometer. Usually, three small dots were marked as close as possible to the cell (typically at a distance 50–200 μm). Commercial MALDI instruments do not have adjustable magnification in their camera systems, and the light source is not very strong either. The white color of the nail polish, which is visible under such circumstances, allows for the quick localization of the cell on the ITO glass.

In our set-up ca. 12–-15 cells might be measured per hour (excluding the time of matrix coating). The number of the cells that could be placed and measured on the ITO glass depends mainly on the manual skills of the technician, who is preparing the sample, but it is advisable to left 4–5 mm space between the cells to be able to mark their position with the nail polish comfortably.

It has to be stressed that Tipp-EX (documents correction fluid) is used in the same way, but for the general marking of the position of the tissue on the ITO glass. Such general localization it too rough for the oocytes to be found under the MALDI camera. Additionally, nail polish is not as dense as Tipp-EX, which makes the marking process easier.

Moreover, white spots allow for marking the region of interest in the software responsible for positioning the area of laser irradiation for MALDI imaging. We noticed that it is a good idea to make a cell imaging along with at least a fragment of a single dot. The material of the nail polish ionizes quite well in the range from hundreds to thousands m/z, giving the peaks of moderate intensities and without significant increase of the background. So, it is possible to observe the nail polish mark along with the signal from the ion of interest (see Fig. 3, white line above the cell). Such “MS” marker allows for estimation of the direction and distance shift between the photography of scanned area and MS-image. This parameter is not very important for bigger objects, like a tissue section, but for a single cell is of higher importance. Available software, allows for positioning image area with limited precision, which is not better than a 10–20 micrometers, so measurement of position shift would give important information during MS-image interpretation.

### Matrix crystallization and a low amount of salt are critical for overall image quality

For single cell experiments, due to the object size, appropriate sample coverage by matrix seems to be the crucial sample preparation step. Pipetting of the matrix solution on the cell is not recommended as uneven crystallization leads to form crystals of various sizes among the sample, with the spaces without crystals (see Fig. 1a). Acquisition of mass spectra, based on such prepared material, might be unsuccessful and usually leads to the sample loss. In our case, the spectra were readable, having overall low quality. So, it is better to use methods based on matrix spraying over the sample surface: smaller matrix droplets form smaller matrix crystals and, as a result, increase in spectrum-to-spectrum repeatability. We used the ImagePrep system, but every instrument (SunCollect by SunChrom, Bruker’s HTX-TM Sprayer, or homemade solutions based on airbrushes) or technology (matrix coverage by sublimation) providing uniform matrix coverage should be fine.

**Fig. 4:**
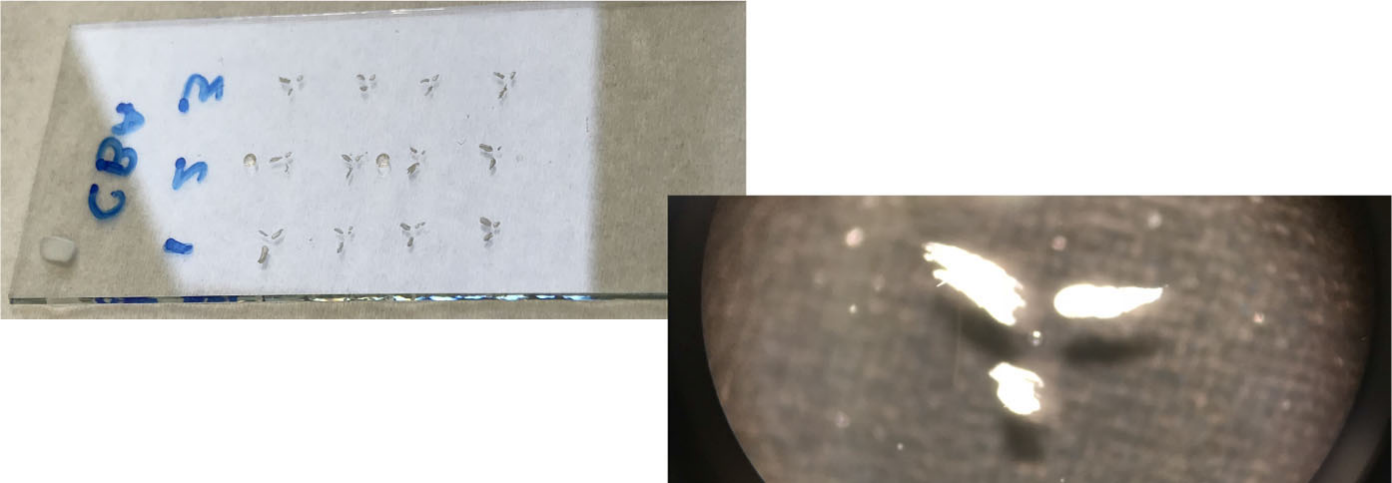
Cells with the nail polish markers deposited on the ITO glass

**Fig. 5:**
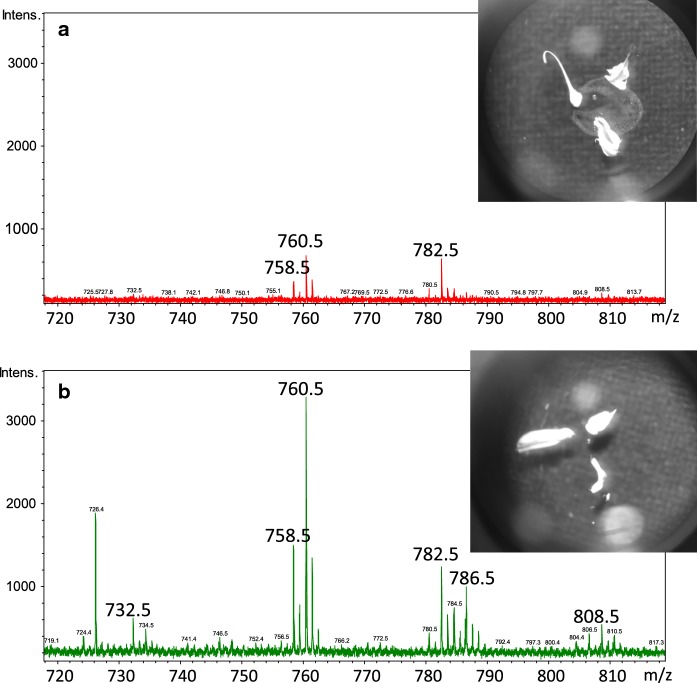
The influence of the salt on the spectra quality. **a** The resulting spectrum from the cell poorly washed from the PBS (clearly visible PBS crystals). **b** The resulting spectrum from the cell washed from the PBS. Both mass spectra have the same intensities on y-axes

It is known that MALDI is more resistant to the presence of salts in the sample than, for example, ESI ion source. Nevertheless, proper washing of the cell from the cell culturing medium is an important step for high-quality spectra. Sometimes simple immerse the ITO glass in the beaker with water for a few seconds helps to obtain better results, especially in the negative ion mode. The spectrum from the cell poorly washed from the PBS, and from the cell prepared appropriately is presented in Fig. 4. It could be clearly seen that in the second case, there are more peaks on the spectrum, and the peak intensity is significantly higher. In our case, during the optimization process we find out that washing the cell before transferring on the ITO glass is sufficient to obtain the spectra of good quality (see “Materials and methods” section) (see Fig. 5).

### Multiple matrix coverage on a single glass slide

**Fig. 6:**
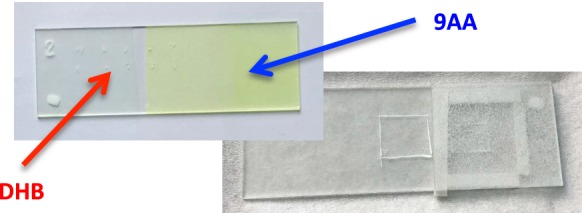
Restricting the area of matrix deposition that allows for multiple matrix coverage at the same ITO glass

Having only a few cells on a single ITO glass designed for the measurement in the positive and negative ion mode, it is a common problem of covering them with the different matrices. We solve this by covering the cells, which should not be sprayed by the particular matrix, with the coverslip. As such coverslip cannot touch the cells on the glass, we used a rolled Parafilm M (Pechiney Plastic Packaging, Chicago, USA), which worked as a weak adhesive gasket to keep a necessary distance (see Fig. 6). After a matrix deposition, coverslip with parafilm can be easily removed, and the measurement with the aid of one matrix could be performed. After the finish of the analysis, another matrix could be applied for the uncovered cells and the analysis in the different mode could be done. This simple procedure can spare ITO glasses, precious biological material, standards for MALDI and a significant amount of time.

### The advantages of the single cell imaging

**Fig. 7:**
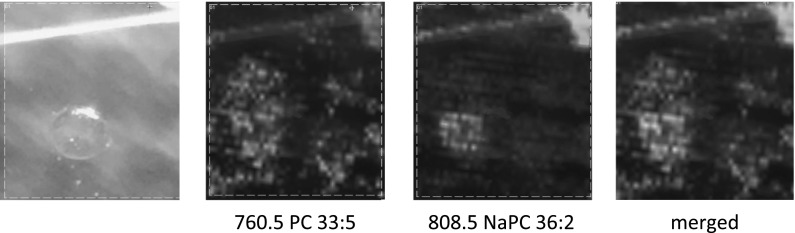
The visualization of localization of the two molecules: m/z 760.5 and m/z 808.5

Single cell imaging might be helpful during the interpretation of obtained results. This kind of analysis may ensure us that the lipids we observe originate directly from the cell. In Fig. 7 we may see the visualization of the distribution of two substances with m/z 760.5 and m/z 808.5. They were both identified as the lipids, but the only NaPC 36:2 (m/z 808.5) was localized in the cell. PC 34:1 (m/z 760.5) was localized outside the cell and might be either an artifact connected with oocyte isolation or might be an effect of the cytoplasm leakage from the cell.

**Fig. 8:**
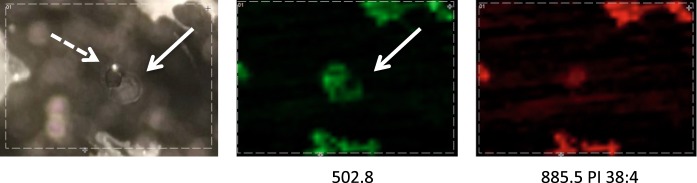
Imperfection in the sample preparation. The cell is marked with the dotted-line arrow and additional substances are marked with the solid-line arrow

Another example can be seen in Fig. 8. In some cases, we detected substances derived from the cells in the area close to the cell in the form of a crown or a leak. We found out that the most probable cause of such a result is the cell damage during the sample preparation step. In such cases cell cytoplasm could be released on the glass surface and dried after a short time. Such observations should be helpful during the refinement of the sample preparation step.

### Looking for the subtle changes

**Fig. 9:**
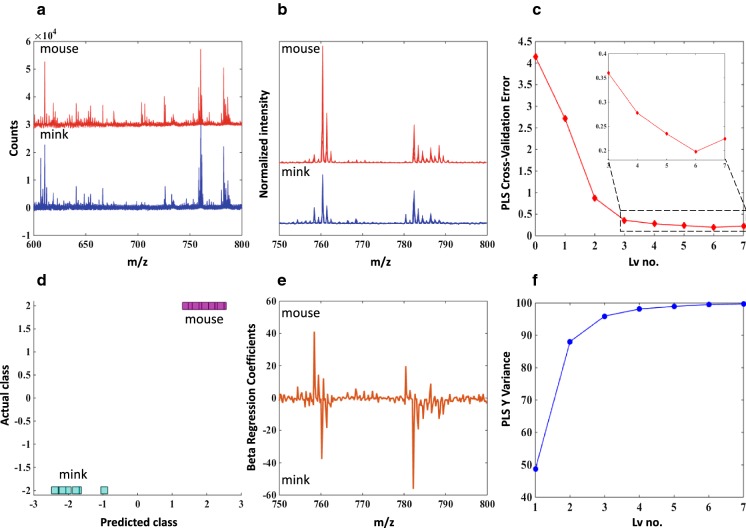
**a** The single spectra of oocytes from C57 mice (red, n = 12) and minks (blue, n = 7) in the lipid region before any preprocessing (only offset for clarity). **b** Preprocessed spectra (binning, baseline, normalization) in the 750–800 m/z range. **c** PLS Cross-Validation (LOO) Error for determination of the correct number of latent variables (Lv). **d** Predicted (x) vs. actual (y) class assignment of the groups. **e** PLS Beta Regression Coefficients showing importance of variables in the discrimination. **f** PLS Y Variance that is covered by a model with a specific number of Lvs

Considering the comparison of oocytes, very often the composition of analyzed samples is different enough to clearly distinguish between cell groups. Such case is observed here, with C57 mice oocytes being slightly different from those of minks. This can be seen in the spectra in Fig. 9, where there are clearly distinguishable signals in the 700–715 m/z range allowing for discrimination. However, when a narrower region is taken into account (to simulate a much harder situation), all of the peaks are present in both groups. Here multivariate techniques can aid, like applied here Partial Least Squares Regression (PLS). PLS is a widely used in spectroscopic field owing its success to properly handling a large number of correlated variables (Mukherjee et al. 2016).

The more new directions (Latent variables—lv.) we include in the model, the better results we will have, as a Y variance increases (Fig. 9, lower right chart). However, incorporating too many may overfit the model and lower the prediction ability. In order to avoid this, a Cross-Validation (CV) approach is used. A minimum of CV error (Fig. 9, upper right chart) indicates the most complex allowed model (Lv. equal 6), while an increase in the CV error with more added Lvs. suggest overfitting (Lv. higher than 6). In our case, considering Y variance and Cross-Validation (CV) approach, the model with 3 lv. is optimal.

To facilitate the understanding of the basis of the discrimination, all Lvs. with corresponding weighting factors are transformed into a single Beta Regression Coefficient (Fig. 9, bottom middle). This represents the way in which different spectral variables (different m/z values) contribute to discrimination. Negative values of peaks correspond to the class assigned a negative value (minks) while positive to the positive class assignment (C57 mice). The higher the absolute value, the higher the importance of the variable.

Our model allows us to indicate the m/z values that discriminates mice and minks oocytes in this narrow—hard to distinguish at first sight—part of the spectrum (for minks 782.5 m/z and for mouse 758.5 m/z). The Area Under the Curve (AUC) is equal to 1 (*p* value = 0.0129 with random permutation testing) for this model thus, we believe that it will be good enough to distinguish subtle changes in a more sophisticated analysis.

## Conclusions

In this article, we presented a different approach for the analysis of single oocytes, which allows for the fast measurement of several samples on a single glass during a single experiment to avoid variability during data acquisition and bias. Additionally, ITO glass allows using two ways of measurement: classical one to measure the lipid profile of the cell and mass spectrometry imaging to obtain lipid localization and draw proper conclusions. In our article, we discussed some tips and tricks that could be useful for the scientists involved in this kind of research. Applying Partial Least Squares Regression (PLS) to the result obtained from mice and minks oocytes in the region hard to distinguish at first sight, and obtaining the discrimination, proved that our approach could be useful in more sophisticated analysis.

## References

[CR1] Aardema H, Lolicato F, van de Lest CHA (2013). Bovine cumulus cells protect maturing oocytes from increased fatty acid levels by massive intracellular lipid storage. Biol Reprod.

[CR2] Auclair S, Uzbekov R, Elis S (2013). Absence of cumulus cells during in vitro maturation affects lipid metabolism in bovine oocytes. Am J Physiol Endocrinol Metab.

[CR3] Bodzon-Kulakowska A, Suder P (2015). Imaging mass spectrometry: instrumentation, applications, and combination with other visualization techniques. Mass Spectrom Rev.

[CR4] Dueñas ME, Essner JJ, Lee YJ (2017). 3D MALDI mass spectrometry imaging of a single cell: spatial mapping of lipids in the embryonic development of zebrafish. Sci Rep.

[CR5] Dunning KR, Russell DL, Robker RL (2014). Lipids and oocyte developmental competence: the role of fatty acids and β-oxidation. Reproduction.

[CR6] Ferreira CR, Saraiva SA, Catharino RR (2010). Single embryo and oocyte lipid fingerprinting by mass spectrometry. J Lipid Res.

[CR7] Ferreira CR, Eberlin LS, Hallett JE, Cooks RG (2012). Single oocyte and single embryo lipid analysis by desorption electrospray ionization mass spectrometry. J Mass Spectrom.

[CR8] Ferreira CR, Jarmusch AK, Pirro V (2015). Ambient ionisation mass spectrometry for lipid profiling and structural analysis of mammalian oocytes, preimplantation embryos and stem cells. Reprod Fertil Dev.

[CR9] Gonçalves RF, Ferreira MS, de Oliveira DN, Canevarolo R, Achilles MA, D’Ercole DL, Bols PE, Visintin JA, Killian GJ, Catharino RR (2016). Analysis and characterisation of bovine oocyte and embryo biomarkers by matrix-assisted desorption ionisation mass spectrometry imaging. Reprod Fertil Dev.

[CR10] González-Serrano AF, Pirro V, Ferreira CR, Oliveri P, Eberlin LS, Heinzmann J, Lucas-Hahn A, Niemann H, Cooks RG (2013). Desorption electrospray ionization mass spectrometry reveals lipid metabolism of individual oocytes and embryos. PLoS ONE.

[CR11] Gulin A, Nadtochenko V, Astafiev A, Pogorelova V, Rtimi S, Pogorelov A (2016). Correlating microscopy techniques and ToF-SIMS analysis of fully grown mammalian oocytes. Analyst.

[CR12] Jasensky J, Boughton AP, Khmaladze A (2016). Live-cell quantification and comparison of mammalian oocyte cytosolic lipid content between species, during development, and in relation to body composition using nonlinear vibrational microscopy. Analyst.

[CR13] Jungheim ES, Macones GA, Odem RR (2011). Associations between free fatty acids, cumulus oocyte complex morphology and ovarian function during in vitro fertilization. Fertil Steril.

[CR14] Kim JY, Kinoshita M, Ohnishi M, Fukui Y (2001). Lipid and fatty acid analysis of fresh and frozen-thawed immature and in vitro matured bovine oocytes. Reproduction.

[CR15] Lapa M, Marques CC, Alves SP (2011). Effect of trans-10 cis-12 conjugated linoleic acid on bovine oocyte competence and fatty acid composition. Reprod Domest Anim.

[CR16] Leão BCS, Rocha-Frigoni NAS, Cabral EC, Franco MF, Ferreira CR, Eberlin MN, Filgueiras PR, Mingoti GZ (2014). Membrane lipid profile monitored by mass spectrometry detected differences between fresh and vitrified in vitro-produced bovine embryos. Zygote.

[CR17] Leroy JLMR, Genicot G, Donnay I, Van Soom A (2005). Evaluation of the lipid content in bovine oocytes and embryos with nile red: a practical approach. Reprod Domest Anim.

[CR18] Leroy JLMR, Vanholder T, Mateusen B (2005). Non-esterified fatty acids in follicular fluid of dairy cows and their effect on developmental capacity of bovine oocytes in vitro. Reproduction.

[CR19] Lolicato F, Brouwers JF, de Lest CHAV (2015). The cumulus cell layer protects the bovine maturing oocyte against fatty acid-induced lipotoxicity. Biol Reprod.

[CR20] Matorras R, Ruiz JI, Mendoza R (1998). Fatty acid composition of fertilization-failed human oocytes. Hum Reprod.

[CR21] Mukherjee P, Lim SJ, Wrobel TP, Bhargava R, Smith AM (2016). Measuring and predicting the internal structure of semiconductor nanocrystals through Raman spectroscopy. J Am Chem Soc.

[CR22] Pirro V, Oliveri P, Ferreira CR, González-Serrano AF, Machaty Z, Cooks RG (2014). Lipid characterization of individual porcine oocytes by dual mode DESI-MS and data fusion. Anal Chim Acta.

[CR23] Polanski Z, Hoffmann S, Tsurumi C (2005). Oocyte nucleus controls progression through meiotic maturation. Dev Biol.

[CR24] Prates EG, Nunes JT, Pereira RM (2014). A role of lipid metabolism during cumulus-oocyte complex maturation: impact of lipid modulators to improve embryo production. Mediat Inflamm.

[CR25] Sanchez-Lazo L, Brisard D, Elis S (2014). Fatty acid synthesis and oxidation in cumulus cells support oocyte maturation in bovine. Mol Endocrinol.

[CR26] Silva-Santos KC, Ferreira CR, Santos GMG (2014). MALDI-MS lipid profiles of oocytes recovered by ovum pickup from Bos indicus and 1/2 indicus × taurus with high vs low oocyte yields. Reprod Domest Anim.

[CR27] Sturmey RG, Reis A, Leese HJ, McEvoy TG (2009). Role of fatty acids in energy provision during oocyte maturation and early embryo development. Reprod Domest Anim.

[CR28] Sudano MJ, Santos VG, Tata A, Ferreira CR, Paschoal DM, Machado R, Buratini J, Eberlin MN, Landim-Alvarenga FDC (2012). Phosphatidylcholine and Sphingomyelin profiles vary in *Bos taurus indicus* and *Bos taurus taurus* in vitro- and in vivo-produced blastocysts. Biol Reprod.

[CR29] Tata A, Sudano MJ, Santos VG, Landim-Alvarenga FDC, Ferreira CR, Eberlin MN (2013). Optimal single-embryo mass spectrometry fingerprinting. J Mass Spectrom.

